# Genetic diversity and population structure of the Asian citrus psyllid in China

**DOI:** 10.1093/jisesa/iead120

**Published:** 2024-01-09

**Authors:** Aijun Huang, Jiayu Ma, Jin Yang, Bo Chen, Jun Zhou, Long Yi

**Affiliations:** College of Life Science, Gannan Normal University, Ganzhou 341000, China; National Navel Orange Engineering Research Center, Ganzhou 341000, China; College of Life Science, Gannan Normal University, Ganzhou 341000, China; College of Life Science, Gannan Normal University, Ganzhou 341000, China; College of Life Science, Gannan Normal University, Ganzhou 341000, China; College of Life Science, Gannan Normal University, Ganzhou 341000, China; National Navel Orange Engineering Research Center, Ganzhou 341000, China; College of Life Science, Gannan Normal University, Ganzhou 341000, China; National Navel Orange Engineering Research Center, Ganzhou 341000, China

**Keywords:** Asian citrus psyllid, citrus Huanglongbing, mitochondrial COI, genetic diversity, demographic history

## Abstract

The Asian citrus psyllid (ACP) is the main vector of Citrus Huanglongbing, the most damaging citrus disease, causing significant financial losses in the citrus industry. Global warming has expanded the habitat of this pest, allowing it to continue its northward migration to China. Population genetic information of ACP is fundamentally essential for species management. This study investigated the genetic diversity and population structure of Chinese ACP using the mitochondrial cytochrome oxidase subunit I gene by dataset comprised 721 sequences from 27 geographic sites in China. Low haplotype diversity (0.323 ± 0.022) and low nucleotide diversity (0.00071 ± 0.00007) were observed in the entire population, which may indicate recent founder events. Twenty-three haplotypes were identified and clustered into 2 haplogroups: haplogroup I and haplogroup II. Haplogroup II included only 2 unique haplotypes, which occurred exclusively in the Southwest China ACP population. Genetic differentiation analyses were also indicative of Southwest China population was significantly differentiated from the remaining populations. Demographic history analysis showed that ACP population in China has experienced demographic expansion. Our results provided a better understanding of the genetic distribution patterns and structures of ACP populations in China.

## Introduction

Asian citrus psyllid (ACP), *Diaphorina citri* Kuwayama (Hemiptera: Liviidae), is regarded as one of the most economically significant citrus pests worldwide (Grafton-Cardwell et al. 2013), not only because it causes severe damage to citrus by direct feeding on leaves, but more importantly it is also the primary vector of Citrus Huanglongbing (HLB), which is an unprecedented challenge posing in citrus production (da Graça et al. 2016). Available evidence suggests that the ACP may have originated in the Indian subcontinent (Hollis 1987) from where it subsequently spread to other tropical and subtropical regions of Asia. It was initially mentioned in Taiwan in 1907 (Kuwayama 1908) and was first recorded in mainland China in the Chaoshan area of Guangdong Province in 1934 (Hoffmann 1936), afterward, it was gradually noticed in several southern Chinese provinces, including Fujian, Guangxi, Sichuan, Jiangxi, Zhejiang, Hunan, Guizhou, and Yunan (Huang 1953, Yao 1985, Wang et al. 2016). Currently, it has happened in over 300 counties across 10 provinces of mainland China. The spread of ACP has promoted the spreading of HLB, causing enormous economic losses in citrus production in China (Zhou 2020). Furthermore, owing to global warming, ACP may continue to migrate northward (Wang et al. 2015), which may result in an increase in the incidence of insect-mediated plant diseases and the expansion of their host range (Merrill et al. 2008, Deutsch et al. 2018, Wu et al. 2020). Therefore, it is urgently needed for efficient management strategies to control both ACP and HLB.

Population structure is a crucial component of population genetics and can broadly be defined as the amount and distribution of genetic diversity within and among populations. Research on population genetic structure enables us to evaluate the levels of gene flow among populations in various geographic areas (Urbanelli et al. 2000), quantify genetic differentiation among populations (Weill et al. 2000), and infer population’s history, including changes in population size, ancestral populations, and range expansions or contractions (Porretta et al. 2007). Such studies can serve to elucidate the basic biology and ecology of insect pest species and, furthermore, aid in the development of efficient control measures.

Particularly, sequence variations in the mitochondrial cytochrome oxidase subunit I (COI) gene region remain one of the most frequently employed genetic markers for conducting genetic structure and diversity analyses (Brown et al. 1979, Oines and Brannstrom 2011). The mitochondrial COI has been utilized to study ACP population structure and diversity in different geographical regions in China. Using this gene marker, Zhang et al. (2019) revealed a notable genetic variation between the population of Southwest China and other regions of southern China. From these observations, they further speculated on the possibility of 2 distinct invasion routes in China: one in Guangdong and the other in Yunnan. In addition, evidence suggests that ACP is still experiencing population expansion, which could be correlated with the extensive citrus cultivation (Meng et al. 2018). However, genetic diversity or structural studies necessitate sampling marked individuals from various environments, which mandates sampling must be extensive to be statistically useful. Nevertheless, data on the ACP genetic diversity and structure in China are inadequate, and further studies are required.

In this study, different geographic ACP samples collected from southern China were analyzed using mitochondrial COI as a molecular marker. The results will provide fundamental genetic information to deepen our understanding of the genetic diversity and structure of ACP in China and also help us further speculate on the origin and diffusion route of ACP in China.

## Materials and Methods

### Psyllid Sampling Collection

**Fig. 1.**
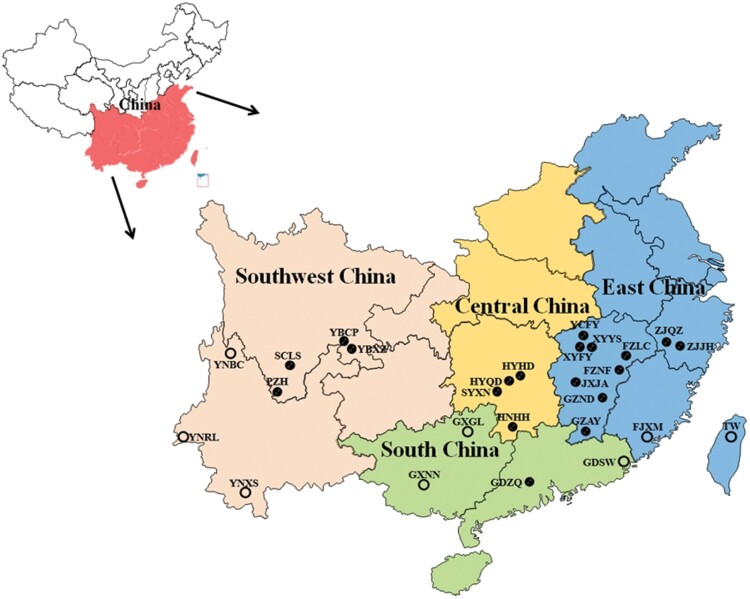
Sampling sites of ACP populations in China. The black solid circles are the sampling locations for this study, and the black hollow circles are locations of the downloaded sequences from GenBank.

Between 2021 and 2022, a total of 570 ACP individuals (adults) were collected from commercial and unmanaged citrus groves at 19 sites in southern China, which is a major ACP occurrence region. The positioning system coordinates of each sampling site were obtained using a portable GPS instrument (Zhuolin A6, Hefei, China). Prior to DNA extraction, the collected specimens were preserved in 75% ethanol and kept at −20°C. In addition, 151 publicly available ACP mitochondrial COI sequences (Wang et al. 2017, Zhang et al 2019) were also contained in our analyses, and their sampling sites were Ruili (*n* = 20), Xishuangbanna (*n* = 24), and Binchuan (*n* = 20) of Yunnan Province, Shanwei (*n* = 20) in Guangdong Province, Guiling (*n* = 20) and Nanning (*n* = 20) in Guangxi Province, Xiamen (*n* = 20) in Fujian Province, and Taiwan (*n* = 17). In all, analyses were conducted in 721 sequences from 27 sampling sites (Table 1). According to geographic origin, all samples were classified into 4 groups: East China, South China, Central China, and Southwest China (Fig. 1).

**Table 1.** Detailed information of ACP samples used in this study

Geographic region	Sampling locations	Geographical coordinates	Plant host	No. of individual	Accession numbers
East China	Yichun, Jiangxi (YCYF)	N28.591° E114.985°	*C. reticulata*	30	OP906166-OP906195
	Yushui, Jiangxi (XYYS)	N27.871° E114.91°	*C. reticulata*	30	OP905956-OP905985
	Fenyi, Jiangxi (XYFY)	N27.857° E116.247°	*C. reticulata*	30	OP905986-OP906015
	Linchuan, Jiangxi (FZLC)	N27.826° E114.595°	*C. reticulata*	30	OP905686-OP905715
	Nanfeng, Jiangxi (FZNF)	N27.223° E116.494°	*C. reticulata*	30	OP905896-OP905925
	Ji’an, Jiangxi (JXJA)	N26.805° E114.945°	*C. reticulata*	30	OP905836-OP905865
	Ningdu, Jiangxi (GZND)	N26.247° E116.141°	*C. sinensis*	30	OP905926-OP905955
	Anyuan, Jiangxi (GZAY)	N25.197° E115.355°	*C. sinensis*	30	OP905866-OP9058995
	Xiamen, Fujian (FJXM)	N24.479° E118.0894°	*Murraya paniculata*	20	MH970714-MH970719
	Jinhua, Zhejiang (ZJJH)	N29.556° E118.826°	*C. reticulata*	30	OP906196-OP906225
	Quzhou, Zhejiang (ZJQZ)	N28.929° E118.797°	*C. reticulata*	30	OP906226-OP906255
	Taiwan (TW)^a^	N23.699° E120.9605°	*Murraya paniculata*	17	KX762326-KX762342
South China	Zhaoqing, Guangdong (GDZQ)	N23.426° E112.540°	*C. sinensis*	30	OPO905716-OP905745
	Shanwei, Guangdong (GDSW)^a^	N22.804° E115.0983°	*Murraya paniculata*	10	KX762380-KX762389
	Guiling, Guangxi (GXGL)^a^	N24.778° E110.4996°	*Clausena*	20	KX762440-KX762459
	Nanning, Guangxi (GXNN)^a^	N22.524° E109.3485°	*Murraya paniculata*	20	KX762460-KX762479
Central China	Xinning, Hunan (SYXN)	N26.537° E110.954°	*C. sinensis*	30	OP906136-OP906165
	Qidong, Hunan (HYQD)	N26.907° E111.624°	*C. sinensis*	30	OP905776-OP905805
	Hengdong, Hunan (HYHD)	N26.998° E113.119°	*C. sinensis*	30	OP905806-OP905835
	Huaihua, Hunan (HNHH)	N27.872° E109.748°	*C. sinensis*	30	OP905746-OP905775
Southwest China	Xuzhou, Sichuan (YBXZ)	N28.625° E104.424°	*C. sinensis*	30	OP906076-OP906105
	Cuiping, Sichuan (YBCP)	N28.770° E104.625°	*C. sinensis*	30	OP906106-OP906135
	Liangshan, Sichuan (SCLS)	N28.157° E103.501°	*C. sinensis*	30	OP906046-OP906075
	Panzhihua, Sichuan (PZH)	N26.555° E101.933°	*C. sinensis*	30	OP906016-OP906045
	Ruili, Yunnan (YNRL)^a^	N24.021° E97.8635°	*C. reticulata*	20	KX762549-KX762568
	Binchuan, Yunnan (YNBC)^a^	N25.827° E100.5751°	*C. reticulata*	20	KX762520-KX762539
	Xishuangbanna, Yunnan (YNXS)^a^	N21.99° E100.79°	*C. reticulata*	24	MH970777-MH970800

^a^COI sequences are downloaded from NCBI.

### DNA Extraction and Sequencing

Whole-body genomic DNA was extracted from individual specimens using the DNeasy Blood and Tissue kit (Qiagen Inc., Valencia, CA, USA) following the manufacturer’s protocol. The quality of the extracted DNA was assessed by NanoDrop 2000c spectrophotometer (Thermo Fisher Scientific). DNA extracts within the A260 nm/A280 nm ratio range of 1.8–2.0 were used for subsequent analyses.

A fragment of the mitochondrial COI gene was amplified by polymerase chain reaction (PCR) using primer sets COI-F (5ʹ-AGGAGGTGGAGACCCAATCT-3ʹ) and COI-R (5ʹ-TCAATTGGGGGAGAGTTTTG-3ʹ). Each PCR reaction was conducted in a 25.0 μl final reaction volume containing 12.5 μl of Premix Taq (TaKaRa), 0.5 μl each of forward and reverse primers (10 μM), 10.5 μl of nuclease-free water, and 1.0 μl of extracted DNA template. The amplification was performed using a Veriti 96-Well thermal cycler (Applied Biosystems, California, USA) with the following cycle conditions: denaturation at 95 °C for 3 min; followed by 35 cycles of 95 °C for 20 s, 56 °C for 20 s, 72 °C for 1 min; and a final elongation step at 72 °C for 10 min. The PCR products were separated on 1.3% agarose and stained with SolarRed (Solarbio, Beijing, China). The target fragments were purified using the DNA purification kit (Solarbio, Beijing, China), according to the manufacturer’s instructions, the purified fragments were delivered to the Suzhou Genewiz Biotech Company (Suzhou, China) for sequencing.

### Data Preparation and Genetic Diversity Analysis

The COI sequences were manually edited using SeqMan 7.1.0 and aligned using ClustalW in MEGA 7. Genetic diversity parameters, including the number of polymorphic sites (S), synonymous (Ks) or nonsynonymous (Ka) substitution rates, haplotype diversity (Hd), number of haplotypes (H), and nucleotide diversity (π) were determined using DnaSP 5.10.01 (Librado and Rozas 2009). When Hd value <0.5 and π value <0.005 suggest a recent population bottleneck or founder event by a single or few lineages; Hd value >0.5 and π value <0.005 suggest recent population bottleneck followed by rapid population growth and accumulation of mutations; Hd value <0.5 and π value >0.005 suggest divergence between geographically subpopulations; Hd value >0.5 and π value >0.005 indicate a large stable population with long evolutionary history or second contact between differentiated lineages (Grant and Bowen 1998).

To evaluate the level of genetic differentiation among ACP populations, pairwise Fst and gene flow (Nm) among the 4 geographic regions (East China, South China, Central China, and Southwest China) were calculated using Arlequin software (Excoffier and Lischer 2010). The Fst value represents the level of differentiation among populations; specifically, 0 < Fst < 0.05 indicates slight genetic differentiation; 0.05 < Fst < 0.15 indicates moderately differentiation; 0.15 < Fst < 0.25 indicates high differentiation, Fst > 0.25 indicates severe differentiation. In turn, the Nm value presents the relative strength of the gene flow among populations. When Nm value <1, a low level of gene flow occurs, whereas when Nm value >1, a high level of gene flow occurs. The overall genetic variance was calculated by analysis of molecular variance (AMOVA) (Excoffier and Lischer 2010).

### Phylogenetic Analysis and Haplotype Network

To understand the phylogenetic relationships among different haplotypes, a neighbor-joining (NJ) tree were constructed with 1,000 bootstrap value using MEGA 7.0 software (Kumar et al. 2018). Haplotype network was visualized by PopART 1.7 (Population Analysis with Reticulate Trees) using the method of Templeton, Crandall, and Sing (TCS) (Clement et al. 2000, Leigh and Bryant 2015).

### Demographic Analysis

The historical demographic expansions were examined by using 2 methods. First, neutral tests, including Tajima’s D (Tajima 1989), Fu and Li’s D, and Fu and Li’s F (Fu 1997) tests, were performed using DnaSP software. Second, mismatch distribution analysis was executed to detect historical population expansion events in Arlequin software. A multimodal pattern reveals that populations are at a demographic equilibrium, whereas a unimodal pattern suggests that populations are experiencing a dramatic demographic expansion. Arlequin software was also used to calculate the sum of the squared deviations (SSDs) between the observed and expected mismatches and the raggedness index (*r*) of the observed distribution. A small *r* value indicated that populations had undergone recent demographic expansions, and a significant value of SSD was an indication of relatively constant population size.

## Results

### Genetic Diversity

**Fig. 2.**
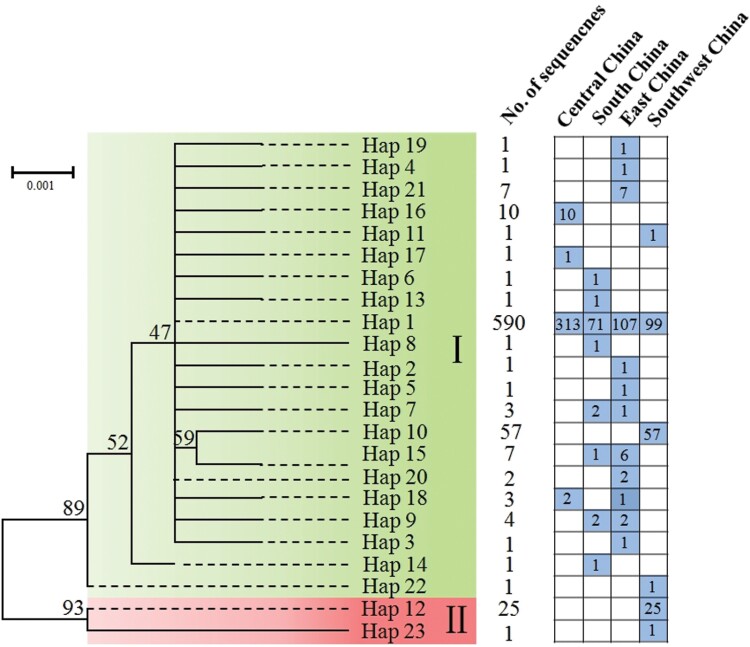
ACP haplotypes phylogenetic analyses and geographic distribution. Bootstrap values are indicated at each node. The distribution and number of haplotypes in different populations are shown in the right panel.

The target COI region (706 bp) was successfully amplified and sequenced for all 570 ACP specimens, and the acquired nucleotide sequences were deposited in GenBank under accession numbers: OP905686-OP906255 (Table 1). A total of 24 polymorphic sites were observed, of which 14 were singleton variable sites and 10 were parsimony informative sites. These polymorphic sites resulted in 23 haplotypes (Hap 1–Hap 23). Among the 23 haplotypes, 5 haplotypes were shared among 2 or more than 2 populations, and 18 haplotypes were restricted to 1 population (Fig. 2). Hap 1 was the most prevalent and widespread, representing 81.8% of all the samples, and occurred in all 4 populations. Hap 10 (7.9%) and Hap 12 (3.5%) were the second and third most frequent haplotypes, respectively, and Hap 12 was exclusively discovered in Southwest China, specifically, only in Xishuangbanna of Yunnan province. Meanwhile, the population in East China shares 3 and 1 common haplotypes with the populations in South China and Central China, respectively.

Low haplotype diversity (0.323) and low nucleotide diversity (0.071) were observed in the entire ACP sample, indicating that populations may have experienced a recent population bottleneck or founder effect. Notably, ACP population from Southwest China exhibited high Hd (0.599) and low π (0.00172), implying that populations from this region may have experienced population bottleneck followed by rapid population growth and mutation accumulation. The remaining populations were with low Hd (0.137–0.213) and π (0.00022–0.00035) values, indicating possible bottleneck effects or founder events in the population history (Table 2). The nonsynonymous (Ka) and synonymous (Ks) rate ratio are used to measure selective pressure at the protein level. The Ka/Ks values for the East, South, and Central China populations were all lower than 1, suggesting that these three populations were predominantly affected by purifying selection, whereas that of Southwest population was greater than 1, indicating that this population was under natural selection.

**Table 2.** Genetic diversity estimates of ACP populations

Populations	*N*	*n*	Ka/Ks	Haplotype	Haplotype diversity (Hd)	Nucleotide diversity (π)
East China	337	12	0.283	Hap1, Hap2, Hap3, Hap4, Hap5, Hap7, Hap9, Hap15, Hap18, Hap19, Hap20, Hap21,	0.137 ± 0.026	0.00022 ± 0.00004
South China	80	8	0.500	Hap1, Hap6, Hap7, Hap8, Hap9, Hap13, Hap14, Hap15,	0.213 ± 0.061	0.00035 ± 0.00011
Central China	120	4	0.029	Hap1, Hap16, Hap17, Hap18	0.199 ± 0.047	0.00029 ± 0.00007
Southwest China	184	6	1.099	Hap1, Hap10, Hap11, Hap12, Hap22, Hap23	0.599 ± 0.023	0.00172 ± 0.00016
All	721	23	0.707	Hap1–Hap23	0.323 ± 0.022	0.00071 ± 0.00007

*N*: number of individuals, *n*: number of haplotypes.

### Haplotype Analysis

Phylogenetic analysis showed that the haplotypes from all populations could be divided into 2 haplogroups (I and II); haplogroup I is present in all populations, whereas haplogroup II consisted exclusively of the haplotypes from the population of Southwest China (Fig. 2).

**Fig. 3.**
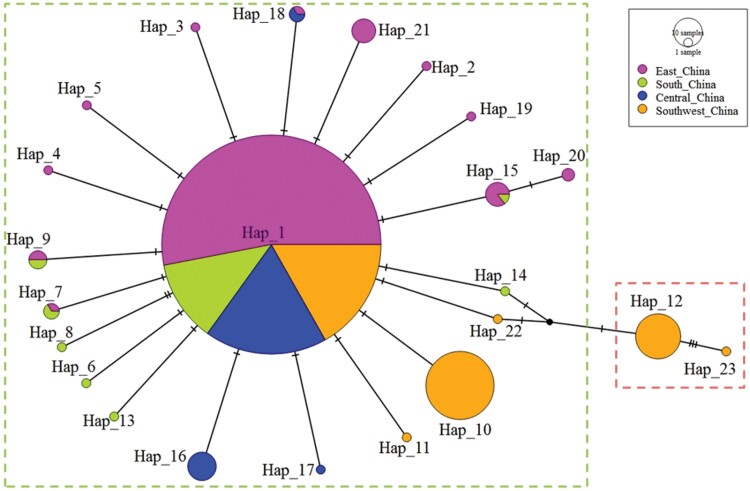
TCS network of ACP based on COI haplotypes. The sizes of the circles indicate the number of the individuals with each given haplotype.

The haplotype network was constructed based on the TCS inference method and was divided into two subdivisions: one included 21 haplotypes (Hap 1—Hap 11, Hap 13—Hap 22), distributed in all four populations, and the other included two haplotypes (Hap 12 and Hap 23) that were only distributed in Southwest China. The pattern of the network was star-like; the highest frequency of Hap 1 occupied a central position, and all other haplotypes arose from it through one or several mutational steps (Fig. 3). Due to its central position in the network, haplotype Hap 1 may be the maternal ancestral haplotype for the ACP populations analyzed herein.

### Genetic Differentiation/Population Structure Analysis

Pairwise Fst tests indicated genetic differentiation among the 4 populations based on the mitochondrial COI gene (Table 3). Particularly, the Fst values among East China, South China, and Central China were lower than 0.05, indicating that these 3 populations were slightly differentiated, whereas the Fst values between Southwest China and the other 3 populations were all >0.15, suggesting a high level of genetic differentiation in the Southwest China ACP population. The pairwise Nm values between the 4 populations were >1, suggesting a high level of gene flow between the pair-member populations. The highest Nm value was found between East China and South China, while the values between Southwest China and the other 3 populations were relatively low (Table 3), suggesting limited gene flow exchange.

**Table 3.** Pairwise Fst (lower left) and gene flow (Nm) (upper right) among different geographic populations of ACP based on mitochondrial COI gene

Geographic population	East China	South China	Central China	Southwest China
East China	—	101.29	6.15	1.11
South China	0.002	—	7.99	1.21
Central China	0.039	0.030	—	1.10
Southwest China	0.183	0.172	0.185	—

The overall nonhierarchical AMOVA test based on all the samples revealed the genetic structure of the populations. AMOVA analysis showed that 16.77% of the genetic variation occurred among populations, with the remaining 83.23% originating from within populations. Genetic variation occurred mainly within the population (Table 4).

**Table 4.** AMOVA for entire ACP samples

Source of variation	*df*	Sum of squares	Variance components	Percentage of variation
Among populations	3	22.404	0.045^*^	16.77
Within populations	717	158.581	0.221^*^	83.23
Fixation index	0.168	*P* = 0.000		

^*^Extremely significant difference (*P* < 0.01).

### Demographic History

**Fig. 4.**
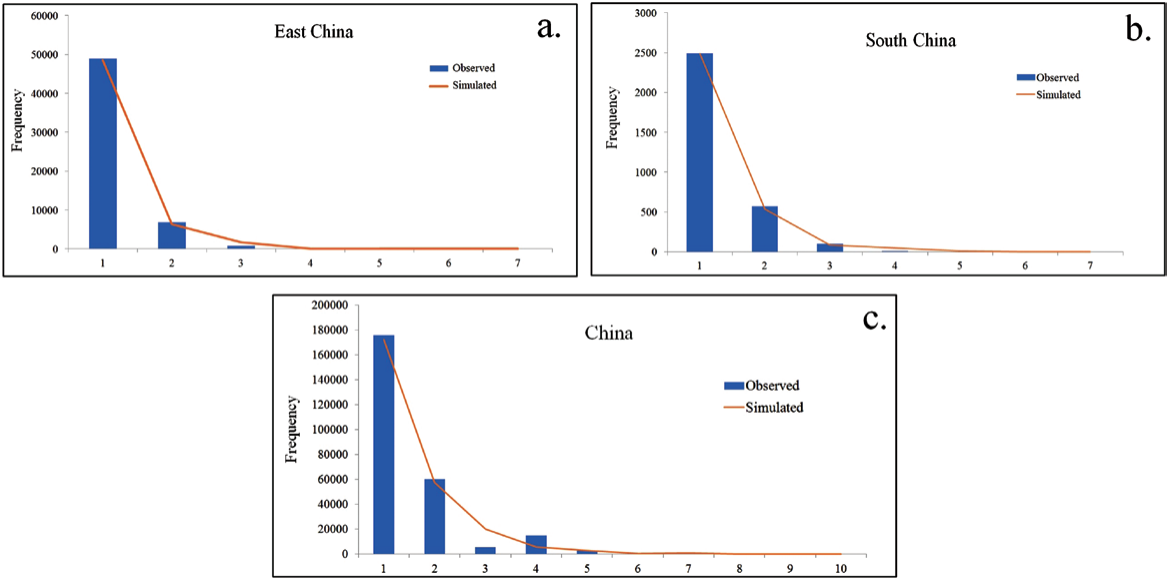
Mismatch distribution of CO1 genes of ACP populations: a) population from East China; b) population from South China; c) population of entire samples.

The neutral test of East China, South China, and the entire sample population showed significantly negative values for Tajima’s D, Fu and Li’s D, and Fu and Li’s F, and reached a significant level (Table 5), indicating that these populations departed from neutral mutation and experienced recent demographic expansion. Furthermore, the mismatch distribution of these 2 populations showed a unimodal profile (Fig. 4), also suggesting that both of the ACP populations may have undergone sudden demographic expansion, and the *r* values are small but SSD values are nonsignificant, both of these were in agreement with the results of neutral tests. The neutral test of the populations from Central China and Southwest China also showed negative values but did not reach a significant level.

**Table 5.** Neutrality test and mismatch distribution analysis of *D. citri* populations

Populations	Neutrality test			Mismatch distribution analysis	
	Tajima’D	Fu & Li’s D	Fu & Li’s F	SSD	*r*
East China	−2.053^*^	−4.120^**^	−4.031^**^	0.000	0.561
South China	−2.126^**^	−3.398^**^	−3.510^**^	0.000	0.391
Central China	−1.105	−0.627	−0.916	0.002	0.403
Southwest China	−0.273	−2.283	−1.876	0.032	0.148
All	−2.124^**^	−5.564^**^	−4.991^**^	0.004	0.247

Tajima’s D, Fu & Li’s D, and Fu & Li’s F: different methods of neutral test.

SSD: sum of the squared deviations.

*r*: raggedness index.

^*^Significant difference (*P* < 0.05),

^**^extremely significant difference (*P* < 0.01).

## Discussion

### Genetic Diversity

Herein, we present a systematic analysis of the ACP population diversity and structure based on the mitochondrial COI gene in China. Specifically, low haplotype diversity and nucleotide diversity were observed in the entire population, implying the population have experienced a recent population bottleneck or founder effect. The findings are consistent with a previous study (Zhang et al. 2019). While, in the previous study, small sample sizes at most of the sampling sites could result in the low diversity observed. In our study, the datasets comprised 721 samples, with 570 samples were collected from 19 locations, each of which contained no less than 30 samples from different orchards. The other 151 sample sequences were selectively downloaded from NCBI. The dataset from each site was carefully controlled to ensure statistical significance. The identical outcomes provide further confirmation of the low haplotype and nucleotide diversity of the ACP population in China based on the COI gene marker.

Phylogenetic and haplotype network analyses suggested the presence of 2 haplogroups: Haplogroup I and Haplogroup II in China. Haplogroup II included only 2 haplotypes; Hap 12 and Hap 23, which were exclusively found in the Southwest population. The analysis of genetic differentiation revealed a significant divergence between the Southwest ACP population and those from East, South, and Central China. These findings are in agreement with previously reported results that obtained with the use of COI gene markers or primary endosymbiont Wang et al. 2017, 2018, Zhang et al. 2019). Based on these results, it has been recommended that 2 introduction events occurred in China, one in Guangdong and the other in Yunnan (Zhang et al. 2019). Although unique haplotypes were identified in the Southwest China population in this or previous studies, Hap 1 was the dominant haplotype in Southwest China population as well as in other populations. Based on the haplotype network, Hap12 and Hap 23, the unique haplotypes identified in Southwest population were also from Hap 1 through several mutation steps, and their parental haplotypes were from the haplogroup I. Combining the gradually reported ACP occurrence history in citrus-growing provinces in China, the hypothesis that ACP entering China was a single introduction event cannot be completely ruled out.

It is worth noting that the ACP population from YNXS was solely consisted by the haplotype Hap12 and was completely distinct from the population composition in other sampling locations. This indicated severe population differentiation in this location. Similar results were also observed by Wu et al. (2018) based on the whole mitochondrial genome sequences, and they found that the analyzed ACP mitogenomes formed 3 major mitochondrial groups, MG1 group solely occurred in Southwest China. Potential explanations could be linked to limited migration ability of ACP, geographical isolation, and local environmental factors. The analysis of gene flow provides partial support for the aforementioned explanation, indicating a relative low gene flow between the Southwest ACP population and other 3 populations. Owing to the unique geographical and environmental factors in the southwestern region of China, such as the Yunnan-Guizhou Plateau, with elevations between 1,000 and 1,500 m, a unique meteorological environment, and with high biodiversity (Yang et al. 2015), the rapid expansion of psyllids after their introduction required adaptation to local environmental conditions, resulting in some unique mutations.

### Demographic History

The entire ACP sample had significantly negative values for neutrality tests and unimodal mismatch distribution, both of these revealed historical population expansion. The population expansion may be influenced by 2 key factors: the expanding of citrus planting area (Meng et al. 2018) and living environment (Yang et al. 2006, Russell et al. 2014, Boina and Bloomquist 2015). China’s citrus planting area has increased from 640 km^2^ in 1980 to 5,668.07 km^2^ in 2019, the rapid expansion of citrus planting led to population expansion of ACP. In addition, higher temperatures and increased precipitation also provided suitable living conditions for ACP (Liu et al. 2016). Thus, expanding host cultivation areas and suitable living environment may positively relate with demographic population expansion of ACP.

## Data Availability

All data are presented in the manuscript.
